# Optimization of Vitrification Protocols for Feline Epididymal Spermatozoa: Impact on Post-Warming Sperm Quality and Fertilizing Potential

**DOI:** 10.3390/ani15131919

**Published:** 2025-06-29

**Authors:** Wirakan Kallayanathum, Larindhorn Udomthanaisit, Theerawat Tharasanit

**Affiliations:** 1 Department of Obstetrics, Gynaecology and Reproductions, Faculty of Veterinary Science, Chulalongkorn University, Bangkok 10330, Thailand; wirakan.mind@gmail.com (W.K.); larin.udtns@gmail.com (L.U.); 2 Center of Excellence for Veterinary Clinical Stem Cells and Bioengineering, Faculty of Veterinary Science, Chulalongkorn University, Bangkok 10330, Thailand

**Keywords:** cryopreservation, feline epididymal sperm, non-permeating cryoprotectant, trehalose, vitrification

## Abstract

The aim of this study was to optimize a permeating cryoprotectant-free vitrification protocol for feline epididymal spermatozoa and evaluate its effects on post-warming sperm quality and fertilizing potential. Four experiments were conducted to assess the impact of the disaccharide concentration (sucrose), type (sucrose vs. trehalose), and stepwise mixing volume with equilibration time and fertilization potential. Post-warming sperm motility, viability, DNA fragmentation, and acrosomal integrity were evaluated. Fertilizing ability was assessed by in vitro fertilization (IVF) and embryo development. The optimal condition—0.5 M trehalose with 20 µL stepwise mixing and no equilibration—resulted in the highest post-warming motility and viability. Vitrified sperm exhibited significantly higher acrosomal damage compared to fresh controls, but DNA fragmentation remained minimal. IVF using vitrified sperm achieved comparable cleavage rates, although blastocyst formation was reduced compared to fresh spermatozoa. These findings confirm the feasibility of vitrification without permeating cryoprotectants and provide a simplified, efficient protocol for feline sperm preservation.

## Simple Summary

Cryopreservation of feline sperm is an important tool for assisted reproduction and conservation programs in domestic and wild felids. This study investigated a simplified, permeating cryoprotectant-free vitrification method using disaccharides (sucrose and trehalose) and evaluated factors influencing post-warming sperm quality. The findings demonstrated that optimized vitrification using 0.5 M trehalose, combined with a rapid mixing protocol, preserved sperm motility and viability and supported fertilization and early embryo development. This approach offered a practical alternative for feline sperm cryopreservation in clinical and field settings.

## 1. Introduction

Cryopreservation is a powerful tool for genetic preservation, assisted reproduction, and the conservation of valuable species. In felines, cryopreservation has been successfully applied to both ejaculated and epididymal spermatozoa, enabling the use of gametes even when natural mating or semen collection is not possible [1,2,3,4,5]. Epididymal sperm cryopreservation in felines is commonly performed using the conventional slow-freezing protocol, which typically yields moderate success. Post-warming sperm viability varies widely, ranging from approximately 30% to 57%, depending on the specific cryopreservation protocols employed [6,7,8,9]. This variation is largely influenced by factors such as the choice of extenders, cryoprotectant agents (CPAs), and freezing techniques. However, the slow-freezing method is time consuming—usually requiring 1 to 2 h.—and carries a high risk of cryoinjury, primarily due to the formation of lethal intracellular ice crystals during the freezing process. Alternatively, vitrification, a rapid freezing method that mitigates ice crystal formation, has gained increasing attention. It offers a simplified and cost-effective alternative to conventional slow freezing and is particularly advantageous in field conditions or time-sensitive scenarios where rapid sample preservation is required [10,11]. Although various extenders and CPAs have been evaluated in conventional slow-freezing protocols for feline sperm [12,13,14], vitrification protocols remain less standardized and poorly characterized, particularly for epididymal sperm. To date, vitrification of sperm in domestic mammals has shown variable outcomes, with post-warming motility rates ranging from approximately 26% in boars (*Sus scrofa*) to over 50% in donkeys (*Equus asinus*) and horses (*Equus caballus*) [15,16,17,18]. These differences are primarily attributed to species-specific sperm physiology, vitrification techniques, and the composition of CPAs, underscoring the need for tailored protocol optimization. In domestic cats (*Felis catus*), ultra-rapid freezing—a form of vitrification—preserved sperm with approximately 50% membrane integrity and up to around 20% motility after warming. However, subsequent embryonic development was limited and did not progress to the blastocyst stage [8,19,20]. To vitrify sperm, permeating CPAs such as DMSO and glycerol are often avoided in sperm vitrification due to their cytotoxicity and potential to induce osmotic stress, especially in species with sperm highly sensitive to membrane disruption [21,22,23]. Instead, non-penetrating CPAs such as sucrose, trehalose, and other polymers are preferred. These disaccharide sugars play a crucial role in sperm vitrification by promoting cell dehydration, preventing intracellular ice formation, and stabilizing membrane structures during ultra-rapid cooling [24,25,26,27]. Previous studies have demonstrated that sucrose-based extenders improve post-thaw motility and acrosome integrity in cat sperm. However, the outcomes remain variable, and protocol conditions are not yet standardized [8,19,20]. Given these limitations, further refinement of vitrification protocols is needed to tailor cryoprotectant concentration and handling conditions to the physiological characteristics of feline epididymal sperm. Therefore, the objective of this study was to optimize the vitrification protocol for feline epididymal sperm by evaluating post-warming sperm quality parameters including motility, viability, DNA fragmentation, acrosome integrity, and fertilization ability by IVF. The findings will support the development of more effective sperm preservation strategies for reproductive management, genetic conservation, and assisted reproductive technologies in both domestic and wild felids.

## 2. Materials and Methods

### 2.1. Animals and Reagents

This study aimed to optimize the vitrification protocol for feline epididymal spermatozoa by evaluating the effects of various experimental conditions. All experiments were performed using pooled sperm samples collected from the caudal epididymis of adult domestic cats (*n* = 120). Each treatment group was biologically replicated at least three times, using independently collected sperm samples pooled from 10 individual cats per replicate. This study was conducted in accordance with the ethical guidelines approved by the Animal Care and Use Committee of Chulalongkorn University (Protocol No. 2531004). All chemical reagents were purchased from Sigma-Aldrich (St. Louis, MO, USA), unless otherwise specified.

### 2.2. Experimental Design

#### 2.2.1. Experiment 1: Effect of Sucrose Concentration on Post-Warming Sperm Quality

To identify the optimal concentration of the non-permeating CPAs, spermatozoa were vitrified in solutions containing 0.25 M, 0.5 M, or 0.75 M sucrose dissolved in sterile water. Prior to vitrification, samples were equilibrated for 10 min at room temperature (26 °C). Post-warming sperm quality was assessed based on motility and viability. The condition yielding the highest quality parameters was selected for subsequent experiments.

#### 2.2.2. Experiment 2: Effect of Disaccharide Type (Sucrose vs. Trehalose) on Post-Warming Sperm Quality

This experiment compared the cryoprotective efficacy of two disaccharides: 0.5 M sucrose and 0.5 M trehalose. The pre-vitrification handling, including a 10 min equilibration at room temperature, followed the same procedure as in experiment 1. After warming, sperm motility and viability were assessed, and the superior disaccharide was selected for further optimization.

#### 2.2.3. Experiment 3: Effects of Stepwise Mixing Volume and Equilibration Time on Post-Warming Sperm Quality

To minimize osmotic shock associated with high concentrations of trehalose, sperm suspensions were gradually mixed with vitrification solution using stepwise additions of 5 µL, 10 µL, or 20 µL, accompanied by gentle vortexing. After mixing, the samples were subjected to either immediate vitrification (0 min equilibration) or a 10 min equilibration at room temperature. Post-warming sperm motility and viability were evaluated to identify the most effective combination of mixing volume and incubation time.

#### 2.2.4. Experiment 4: Assessment of Fertilizing Potential Following Optimized Vitrification Protocol

The fertilizing ability of spermatozoa vitrified under optimized conditions was evaluated via IVF using in vitro matured oocytes. Embryo development and quality were then assessed to determine the functional competence of the post-warming spermatozoa.

### 2.3. Feline Epididymal Sperm Collection

Epididymal spermatozoa were obtained from the caudal epididymis of adult domestic cats undergoing routine orchiectomy. Testes were donated with permission from the Neutering Center of the Rabies Control Group, Veterinary Public Health Division, Bangkok Metropolitan Administration. The caudal epididymis was dissected in 0.9% (*w*/*v*) normal saline solution. Spermatozoa were recovered by gently squeezing the caudal epididymis using sterile forceps into pre-warmed (37 °C) holding medium (HM) and then pooled and filtered through a 40 µm nylon mesh to remove tissue debris. No sperm selection method was applied in this study. Initial sperm motility and concentration were determined using a hemocytometer under phase-contrast microscopy. The HM consisted of HEPES-buffered Medium 199 supplemented with 1 mM sodium pyruvate, 2 mM L-glutamine, 100 IU/mL penicillin, 100 μg/mL streptomycin, and 4 mg/mL bovine serum albumin (BSA; embryo-tested grade).

### 2.4. Vitrification and Warming

Vitrification solutions containing disaccharides (e.g., 0.25 M, 0.5 M, and 0.75 M sucrose or 0.5 M trehalose) were prepared separately and mixed with sperm suspensions during each experiment. For all experiments, sperm fractions were adjusted to a final concentration of 10 × 10^6^ sperm/mL in HM before being mixed with vitrification solution in a stepwise manner using aliquots of 5, 10, or 20 μL. The sperm suspension was combined with an equal volume of vitrification solution (200:200 μL per replicate, 1:1 ratio) and equilibrated at room temperature (26 °C) for either 0 or 10 min. The osmolality of vitrification solutions containing 0.5 M sucrose and 0.5 M trehalose was approximately 532 and 538 mOsm/kg, respectively, measured using a freezing-point osmometer (The Fiske^®^ 210, Advanced instruments, Norwood, MA, USA).

Following equilibration, 30 μL aliquots of the sperm-vitrification mixture were dropped from a height of approximately 5 cm directly into liquid nitrogen (LN_2_) using a micropipette. The vitrified droplets rapidly solidified upon contact and were immediately stored in cryovials submerged in LN_2_ at −196 °C for a minimum of 24 h.

For warming, ten vitrified sperm pellets were retrieved and immersed directly into 4 mL of pre-warmed (38.5 °C) holding medium (HM). The suspension was gently agitated for 1 min to allow complete rehydration and warming. Subsequently, the sperm suspension was centrifuged at 800× *g* for 5 min at room temperature. The cell pellet was finally resuspended in 50 μL of HM for sperm evaluation or used for IVF.

### 2.5. Sperm Quality Evaluation

Post-warming sperm quality was evaluated based on motility, viability, DNA integrity, and acrosomal status.

Progressive motility: The proportion of spermatozoa with progressive motility was subjectively assessed. A 10 μL aliquot of sperm sample was placed on a pre-warmed slide and covered with a pre-warmed cover slip and subjectively assessed on a phase-contrast microscope at 200× magnification.

Viability: Sperm viability was evaluated using the membrane-permeable fluorescent dye calcein acetoxymethyl ester (Calcein AM) and the membrane-impermeable nuclear stain ethidium homodimer-1 (EthD-1). This dual-staining method differentiates live and dead sperm based on plasma membrane integrity. Briefly, 10 µL of sperm suspension was incubated with 10 µL of a staining solution containing 2 µM Calcein AM and 4 µM EthD-1 in phosphate-buffered saline (PBS). The samples were incubated in the dark at 37 °C for 15 min. After incubation, 5 µL of the stained sperm suspension was placed on a clean glass slide, covered with a coverslip, and immediately examined under a fluorescence microscope equipped with appropriate filters (excitation/emission: 495/515 nm for Calcein AM and 528/617 nm for EthD-1) at 400× magnification. Live spermatozoa exhibiting intact plasma membranes fluoresced green due to Calcein AM conversion by intracellular esterases, whereas non-viable sperm with compromised membranes showed red fluorescence from EthD-1 nuclear staining. At least 200 spermatozoa per sample were counted to determine the percentage of viable and non-viable cells.

DNA integrity: DNA fragmentation was evaluated using the terminal deoxynucleotidyl transferase dUTP nick-end labeling (TUNEL) assay. Sperm suspensions were fixed in 4% paraformaldehyde for more than 30 min. Following fixation, sperm were permeabilized using 0.1% (*v*/*v*) Triton X-100 in PBS for 5 min. DNA fragmentation was detected using the In Situ Cell Death Detection Kit (Roche, Mannheim, Germany), following the manufacturer’s instructions. Briefly, sperm samples were incubated with the TUNEL reaction mixture (TdT enzyme: label solution = 1:10, *v*/*v*) for 1 h at 37 °C in a humidified chamber. After TUNEL labeling, nuclei were counterstained with 4 µM EthD-1. Spermatozoa showing bright green fluorescence were classified as TUNEL positive, indicating DNA fragmentation or apoptosis, whereas those lacking green fluorescence were considered TUNEL negative. The positive control of DNA strand break was established by treating sperm with 1 mg/mL DNase I for overnight prior to TUNEL staining. At least 200 spermatozoa per sample were counted to determine the percentage of DNA integrity.

Acrosome integrity: Acrosomal integrity was evaluated using fluorescein isothiocyanate-labeled peanut agglutinin (FITC-PNA; Arachis hypogaea) staining. A 10 µL aliquot of sperm suspension was mixed with 10 µL of EthD-1 and incubated at 37 °C for 15 min. Subsequently, 5 µL of the mixture was smeared onto a clean glass slide and air-dried. The samples were fixed in 95% ethanol for 30 s and allowed to air dry again. Acrosomal labeling was performed by applying 50 µL of FITC-PNA solution 0.1 mg/mL to each slide, followed by incubation in a dark, humidified chamber at 4 °C for 30 min. The slides were then rinsed with cold PBS and air-dried. Spermatozoa were examined under a fluorescence microscope at 1000× magnification and classified into three categories based on acrosomal fluorescence: (A) acrosome intact—uniform bright fluorescence over the acrosomal cap; (B) acrosome partially damaged—irregular or fragmented fluorescence, indicating partial loss or abnormality of the acrosomal structure; and (C) acrosomal loss—absence of fluorescence, indicating complete acrosomal exocytosis. At least 200 spermatozoa per sample were counted to determine the percentage of acrosome integrity.

### 2.6. In Vitro Production of Feline Embryos

The ovaries were collected from adult queens (*n* = 30) undergoing routine ovariohysterectomy at the Neutering Center of the Rabies Control Group. In vitro production of feline embryos was conducted in three stages: maturation, fertilization, and embryo culture. The cumulus–oocyte complexes (COCs) were released from the ovaries by repeating slices in HM. Groups of 30–40 COCs were matured for 24 h in in vitro maturation (IVM) medium based on sodium bicarbonate-buffered Medium 199 (M199). Matured oocytes were considered mature based on the presence of expanded cumulus cells and uniform cytoplasm and then randomly distributed to the fresh and vitrified sperm groups for IVF. Following IVM, IVF was performed by co-incubating COCs with spermatozoa at a final concentration of 0.5 × 10^6^ sperm/mL in 50 μL droplets of synthetic oviductal fluid (SOF) supplemented with 4 mg/mL bovine serum albumin (IVC-1). On Day 2 post-IVF, cleaved embryos were transferred to SOF medium supplemented with 10% (*v*/*v*) fetal bovine serum (FBS; Gibco^®^, Invitrogen, Carlsbad, CA, USA) for further culture. All in vitro procedures were carried out at 38.5 °C in a humidified atmosphere containing 5% CO_2_.

### 2.7. Assessment of Embryo Developmental Rate and Quality

Embryo development and morphology were monitored daily using an inverted microscope. Embryos were classified as cleaved on Day 2 (at least 2 cells) and as blastocysts (defined as embryos containing >50 cells with visible blastocoel formation) on Day 7. The percentages of cleaved embryos and blastocysts were evaluated on Days 2 and 7 of culture. For total blastocyst cell count, blastocysts were fixed in 4% paraformaldehyde (PF) and stained with 4′,6-diamidino-2-phenylindole (DAPI) for nuclear visualization under fluorescence microscopy.

### 2.8. Statistical Analysis

Data were analyzed using SPSS program Version 29 (IBM Corp., Armonk, NY, USA). Numerical data, including sperm quality parameters and total cell numbers of blastocysts, were assessed using one-way ANOVA followed by Tukey’s post hoc test. Differences in acrosome intactness, DNA integrity, cleavage, and blastocyst formation rates among experimental groups were assessed using the Chi-square test. Data are presented as the mean ± standard deviation (SD), and 95% confidence intervals were calculated. In all cases, differences were considered significant when *p* < 0.05.

## 3. Results

### 3.1. Experiment 1: Effect of Sucrose Concentration on Quality of Vitrified Warmed Feline Epididymal Sperm

The effects of different sucrose concentrations on the motility and viability of feline epididymal spermatozoa before and after vitrification are summarized in Table 1. Prior to vitrification, no significant differences were observed in sperm motility and viability among the groups, with all treatments showing consistently high values. Pre-vitrified motility was highest in the 0.25 M and 0.5 M sucrose groups (80%), while the 0.75 M sucrose group showed slightly lower motility (62.5 ± 5%). After vitrifying and warming, sperm motility and viability were significantly reduced in all treatment groups (*p* < 0.05). Although there were no significant differences among vitrification media used, the 0.5 M sucrose group demonstrated the highest post-warming motility (10%) and viability (13.88 ± 2.47%) compared to the 0.25 M and 0.75 M sucrose groups.

### 3.2. Experiment 2: Effect of Disaccharide Type (Sucrose vs. Trehalose) on Quality of Vitrified Warmed Feline Epididymal Sperm

Building on the results from experiment 1, where 0.5 M sucrose was identified as the most effective concentration, the comparison of sucrose versus trehalose was performed. As shown in Table 2, pre-vitrified sperm motility and viability were identical between groups (80% motility and 96.12 ± 1.19% viability), confirming consistent baseline quality across treatments. After vitrification and warming, sperm motility and viability significantly declined in both groups. The trehalose group demonstrated slightly better post-warming results, with motility maintained at 10% and viability at 18.92 ± 5.70%, compared to the sucrose group, which showed 8.33 ± 2.58% motility and 15.95 ± 4.95% viability.

### 3.3. Experiment 3: Effects of Stepwise Mixing Volume and Equilibration Time on Quality of Vitrified Warmed Feline Epididymal Sperm

To further investigate the vitrification protocol, experiment 3 evaluated the effects of stepwise mixing volume and equilibration time on post-warming sperm quality, using 0.5 M trehalose in the vitrification solution. After 1:1 mixing with the sperm fraction, the final trehalose concentration was adjusted to 0.25 M. As shown in Table 3, all groups had equal baseline sperm quality, with pre-vitrified motility fixed at 80% and pre-vitrified viability ranging from 95.08 ± 1.52% to 96.48 ± 1.66%. Post-warming results showed that larger mixing volumes were associated with improved sperm motility and viability, regardless of the equilibration time. The highest post-warming motility (21.25 ± 6.29%) and viability (32.15 ± 8.42%) were observed in the group treated with a 20 µL drop and 0 min equilibration, followed by the same volume with 10 min equilibration (13.75 ± 2.5% motility and 22.3 ± 8.66% viability). In contrast, the lowest post-warming outcomes were recorded in the 5 µL drop groups, particularly for motility (8.75 ± 4.79% and 10 ± 4.08%) and viability (14.5 ± 7.85% and 14.13 ± 7.20%) at 0 and 10 min, respectively. Notably, 10 µL mixing volumes produced intermediate results, with post-warming motility ranging from 11.25 ± 2.5% to 12.5 ± 2.89% and viability between 19.73 ± 9.40% and 19.95 ± 8.90%.

### 3.4. Experiment 4: Effects of Optimized Vitrification Protocol on Fertilizing Potential of Vitrified Warmed Feline Epididymal Sperm

Building on the previous optimization of trehalose concentration and handling conditions, experiment 4 assessed the fertilizing potential of spermatozoa vitrified under the most effective protocol. To determine whether sperm damage contributed to the observed differences in embryo development, acrosome status and DNA integrity were further examined. As shown in Table 4, a significant difference in acrosome intactness was found between groups. The control group (fresh epididymal sperm) showed a high percentage of intact acrosomes (84.81 ± 4.22%), with only 9.31 ± 2.22% partially damaged and 4.29 ± 4.01% completely lost. In contrast, sperm in the vitrified group exhibited a significantly lower percentage of intact acrosomes (22.86 ± 3.33, *p* < 0.01) and a much higher rate of partial damage (65.04 ± 1.64, *p* < 0.01). However, no significant difference was found in the proportion of sperm exhibiting complete acrosomal loss between the two groups (12.10 ± 2.02, *p* > 0.05).

In addition, DNA integrity was assessed using the TUNEL assay to examine potential genomic damage induced by vitrification. DNA fragmentation was significantly higher in the vitrified sperm group compared to the control. Fresh epididymal sperm showed minimal DNA damage (0.33 ± 0.29%), whereas sperm from the vitrified group exhibited an elevated level of DNA fragmentation, although the difference was not statistically significant (2.35 ± 1.23%, *p* > 0.05).

To assess the fertilizing capacity of spermatozoa preserved using the optimized vitrification protocol (0.5 M trehalose), IVM and IVF assays were performed. The cleavage rate, blastocyst rate, and total blastocyst cell number were compared between the sperm vitrified in 0.5 M trehalose and fresh epididymal sperm (control group). As shown in Table 5, the average cleavage rate in the treatment group was 68.35 ± 14.16%, which was slightly higher than the control group (61.55 ± 11.41%). However, the blastocyst formation rate was lower in the treatment group (38.5 ± 21.73%) compared to the control (51.03 ± 12.91%), although the difference was not statistically significant. The average total blastocyst cell number did not differ significantly between groups, with 540.88 ± 289.43 cells in the control group and 522.28 ± 335.31 cells in the treatment group.

## 4. Discussion

Effective vitrification of epididymal sperm is critical for conserving feline genetic resources and supporting assisted reproductive technologies (ARTs), especially in endangered felids. This study demonstrates that vitrification without permeating CPAs is feasible by using only a non-permeating disaccharide as the primary extracellular CPA. The disaccharide sugar promotes rapid osmotic dehydration, minimizing intracellular ice formation, and has been shown to be beneficial for sperm cryopreservation in various species [20,28,29,30]. However, its concentration must be optimized to balance dehydration with osmotic stress. Among the tested concentrations, 0.5 M sucrose yielded the highest post-warming motility and viability. Lower concentrations (0.25 M) likely led to insufficient dehydration, increasing ice formation risk, whereas higher concentrations (0.75 M) may have caused over-dehydration and membrane damage. This aligns with the osmotic tolerance limits of feline sperm (around 450–600 mOsm/kg) [31,32]. Importantly, pre-vitrification motility did not vary significantly across treatments, suggesting no immediate functional impairment. Nevertheless, sublethal damage, including alterations in membrane lipids and proteins, may occur during cryopreservation [33,34,35,36,37]. In a subsequent experiment, trehalose (0.5 M) outperformed sucrose at the same concentration. Trehalose likely stabilizes membranes by interacting with phospholipids, replacing water molecules, improving membrane permeability and reducing oxidative stress and ice formation [24,26,38,39,40]. These findings are consistent with studies in other mammalian sperm, where trehalose has been shown to positively protect sperm quality [41,42,43]. To mitigate osmotic shock, we also examined the effects of droplet volume and equilibration time in vitrification medium. A 20 μL droplet improved post-warming sperm quality compared to smaller volumes. However, a 10 min incubation reduced viability, likely due to prolonged hyperosmotic exposure. The estimated equilibration times for a 20 μL droplet was 1.6 min, significantly shorter than 6.7 min for a 5 μL droplet. This suggests that sperm rapidly respond to osmotic stress and prolonged exposure to anisosmotic conditions is detrimental to sperm survival [31,32,44,45,46]. We further evaluated acrosome and DNA integrity. While DNA remained largely intact, acrosome integrity was significantly compromised in vitrified warmed sperm, consistent with previous reports on cryoinjury vulnerability. Sperm DNA is inherently resilient to cryopreservation-induced damage due to its highly compact chromatin structure, which is stabilized by protamines and extensive disulfide bonding [47,48]. Although not directly measured in this study, previous reports have suggested that acrosomal damage may be linked to an increase in cytoplasm Ca^2+^ levels [49,50], reactive oxygen species (ROS) generation, and lipid peroxidation [51,52,53]. While vitrified-warmed sperm exhibited significantly reduced acrosomal integrity, DNA integrity and cleavage rates remained unaffected. However, the observed impairment in blastocyst development is likely attributable to sublethal cryo-induced alterations, including oxidative damage to sperm proteins or lipids, disrupted epigenetic regulation (such as aberrant DNA methylation or faulty protamine-histone exchange), and the loss of essential paternal RNAs or centrosomal components required for zygotic genome activation and sustained embryonic development [49,50,51,52,53]. These subtle molecular disruptions, despite the absence of overt DNA fragmentation, may compromise embryonic competence beyond the cleavage stage. Importantly, we report for the first time that blastocyst development can be achieved using vitrified-warmed sperm without penetrating CPAs. However, while the cleavage rate was similar to controls, blastocyst formation was slightly lower in the vitrified group. This suggests that later embryonic development may be impaired by undetected sperm damage. In this study, vitrified sperm exhibited a sevenfold increase in DNA fragmentation compared to fresh sperm. This finding is consistent with earlier reports suggesting that increased sperm DNA damage, primarily caused by oxidative stress during cryopreservation, can reduce embryo viability and impair developmental progression [54,55]. Additionally, previous studies have proposed that the potential mechanisms include mitochondrial dysfunction [56] and epigenetic disruptions that interfere with embryonic genome activation during the maternal-to-zygotic transition, which may contribute to developmental failure [57,58,59]. Although post-warming motility and viability were modest (21.25% and 32.15%, respectively), they were sufficient to support successful IVF, as demonstrated by cleavage rates comparable to fresh sperm and progression to the blastocyst stage. For artificial insemination, vitrified-warmed sperm can be used. However, intrauterine insemination is recommended for optimal results. These results align with previously reported values (~22% motility and 45% membrane integrity) [8], although lower motility and higher viability (~15% and 57%, respectively) have been observed in felines using sucrose-based extenders [20]. These differences may reflect variations in the sperm source, cryoprotectant composition, and vitrification protocols. We note that the observed variability in blastocyst cell numbers likely reflects inter-donor biological differences and embryo sensitivity to cryo-induced sperm damage. While IVF and early embryo development provide strong indicators of fertilizing potential, future studies involving artificial insemination and embryo transfer are essential to confirm the full-term developmental competence of embryos derived from vitrified sperm. Although pooling sperm reduced intra-replicate variability, it also limited the assessment of individual biological variation. While the total sample size provided valuable data, power analysis suggests larger group sizes are needed to detect moderate effects. Future studies should expand sample size and include individual-level analyses to strengthen biological inference and statistical reliability regarding vitrification outcomes.

## 5. Conclusions

In conclusion, this study highlights the feasibility of cryoprotectant-free sperm vitrification and the importance of optimizing osmotic conditions and handling protocols. Trehalose yielded superior post-warming motility and viability compared to sucrose, supporting its potential as the preferred non-permeating cryoprotectant. While the results are promising, the protocol should be considered preliminary for clinical application. Future studies should incorporate sensitive assays for sperm functionality, such as mitochondrial membrane potential assessment and oxidative stress markers, and consider embryo transfer to evaluate developmental competence more comprehensively.

## Figures and Tables

**Table 1:** The effect of different concentrations of sucrose on sperm quality following vitrification and warming of feline epididymal sperm.

Solution	Pre-Vitrified Motility (%)	Post-Warming Motility (%)	Pre-Vitrified Viability (%)	Post-Warming Viability (%)
Sucrose 0.25 M	80.0 ± 0 ^a^	5.0 ± 0 ^b^	95.63 ± 1.19 ^a^	6.38 ± 1.19 ^b^
Sucrose 0.5 M	80.0 ± 0 ^a^	10.0 ± 0 ^b^	95.63 ± 1.19 ^a^	13.88 ± 2.47 ^b^
Sucrose 0.75 M	62.5 ± 5 ^a^	2.5 ± 2.89 ^b^	95.63 ± 1.19 ^a^	6.25 ± 1.91 ^b^

Superscript letters indicate statistically significant differences between pre- and post-vitrification values within each treatment group (*p* < 0.05).

**Table 2:** The comparison between sucrose and trehalose on sperm quality following vitrification and warming of feline epididymal sperm.

Solution	Pre-Vitrified Motility (%)	Post-Warming Motility (%)	Pre-Vitrified Viability (%)	Post-Warming Viability (%)
Sucrose 0.5 M	80.0 ± 0 ^a^	8.33 ± 2.58 ^b^	96.12 ± 1.19 ^a^	15.95 ± 4.95 ^b^
Trehalose 0.5 M	80.0 ± 0 ^a^	10.0 ± 0 ^b^	96.12 ± 1.19 ^a^	18.92 ± 5.70 ^b^

Superscript letters indicate statistically significant differences between pre- and post-vitrification values within each treatment group (*p* < 0.05).

**Table 3:** Effects of stepwise mixing volume and equilibration time using 0.5 M trehalose in holding medium on post-warming sperm quality of feline epididymal spermatozoa.

Stepwise Mixing Volume	Equilibration Time	Pre- Vitrified Motility (%)	Post-Warming Motility (%)	Pre- Vitrified Viability (%)	Post-Warming Viability (%)
5 µL drop	0 min	80.0 ± 0 ^a^	8.75 ± 4.79 ^b^	96.38 ± 1.49 ^a^	14.5 ± 7.85 ^b^
	10 min	80.0 ± 0 ^a^	10.0 ± 4.08 ^b^	96.3 ± 2.56 ^a^	14.13 ± 7.20 ^b^
10 µL drop	0 min	80.0 ± 0 ^a^	12.5 ± 2.89 ^b^	95.08 ± 1.52 ^a^	19.73 ± 9.40 ^b^
	10 min	80.0 ± 0 ^a^	11.25 ± 2.5 ^b^	96.3 ± 2.56 ^a^	19.95 ± 8.90 ^b^
20 µL drop	0 min	82.5 ± 5 ^a^	21.25 ± 6.29 ^b^	96.75 ± 1.64 ^a^	32.15 ± 8.42 ^b^
	10 min	80.0 ± 0 ^a^	13.75 ± 2.5 ^b^	96.3 ± 2.56 ^a^	22.3 ± 8.66 ^b^

Superscript letters indicate statistically significant differences between pre- and post-vitrification values within each treatment group (*p* < 0.05).

**Table 4:** Comparison of acrosome intactness between fresh and vitrified feline epididymal sperm.

Sperm	Intact (%)	Partial Damage (%)	Complete Loss (%)
Fresh (Control)	84.81 ± 4.22 ^a^	9.31 ± 2.22 ^a^	4.29 ± 4.01 ^a^
Vitrified (Treatment)	22.86 ± 3.33 ^b^	65.04 ± 1.64 ^b^	12.10 ± 2.02 ^a^

Superscript letters indicate statistically significant differences between control and treatment groups within the same parameter (*p* < 0.01).

**Table 5:** Comparing fertilization potential of fresh and vitrified sperm under optimal condition (0.5 M trehalose).

Sperm	Total Oocyte (*N*)	Cleavage (%)	Blastocyst (%) *	Total Blastocyst Cell Number
Fresh (Control)	51	61.55 ± 11.41 ^a^	51.03 ± 12.91 ^a^	540.88 ± 289.43 ^a^
Vitrified (Treatment)	69	68.35 ± 14.16 ^a^	33.97 ± 9.21 ^a^	522.28 ± 335.31 ^a^

Superscript letters indicate statistically significant differences between control and treatment groups within the same parameter (*p* < 0.05). * Embryo development in relation to cleaved embryos.

## Data Availability

Dataset available on request from the authors.

## Abbreviations

The following abbreviations are used in this manuscript:

ARTs	Assisted Reproductive Technologies
BSA	Bovine Serum Albumin
COC	Cumulus–Oocyte Complex
DAPI	4′,6-Diamidino-2-Phenylindole
DMSO	Dimethyl Sulfoxide
EthD-1	Ethidium Homodimer-1
FITC-PNA	Fluorescein Isothiocyanate–Peanut Agglutinin
HM	Holding Medium
ICSI	Intracytoplasmic Sperm Injection
IVF	In Vitro Fertilization
IVM	In Vitro Maturation
LN_2_	Liquid Nitrogen
PBS	Phosphate-Buffered Saline
ROS	Reactive Oxygen Species
SEM	Standard Error of the Mean
SOF	Synthetic Oviductal Fluid
TUNEL	Terminal Deoxynucleotidyl Transferase dUTP Nick-End Labeling
